# High-fat diet feeding exacerbates HIV-1 rectal transmission

**DOI:** 10.1128/msystems.01322-23

**Published:** 2024-02-02

**Authors:** Saroj Chandra Lohani, Amanda E. Ramer-Tait, Qingsheng Li

**Affiliations:** 1 School of Biological Sciences, University of Nebraska-Lincoln, Lincoln, Nebraska, USA; 2 Nebraska Center for Virology, University of Nebraska-Lincoln, Lincoln, Nebraska, USA; 3 Department of Food Science and Technology, University of Nebraska-Lincoln, Lincoln, Nebraska, USA; 4 Nebraska Food for Health Center, University of Nebraska-Lincoln, Lincoln, Nebraska, USA; Duke University School of Medicine, Durham, North Carolina, USA

**Keywords:** HIV-1 rectal transmission, HFD, MSM, inflammatory markers, gut microbial dysbiosis, double humanized BLT mice

## Abstract

High-fat diet (HFD) is well known to impact various aspects of gut health and has been associated with many diseases and inflammation. However, the impact of HFD feeding on HIV-1 rectal transmission has not yet been well addressed. With an increasing threat of HIV-1 infection in men who have sex with men (MSM), where the rectal route is the primary mode of infection, it is imperative to understand the impact of HFD on gut microbiota and inflammation and consequently, its effect on HIV-1 rectal transmission. Here, we utilized our double humanized bone marrow, liver, thymus (dHu-BLT) mouse model to assess the impact of HFD feeding on the host’s susceptibility to HIV-1 rectal transmission. We found that feeding an HFD successfully altered the gut microbial composition within 3 weeks in the dHu-BLT mouse model. In addition, levels of inflammatory mediators, specifically IL-12p70, IP-10, ICAM-1, and fecal calprotectin, were significantly higher in HFD-fed mice compared to control mice on a regular chow diet. We also observed that significantly different inflammatory markers (IL-12p70 and ICAM-1) were negatively correlated with the number of observed ASVs, Shannon diversity, and Faith’s diversity in the HFD-fed group. Notably, when repeatedly challenged with a low dose of HIV-1 *via* a rectal route, mice receiving an HFD were significantly more susceptible to HIV-1 rectal infection than control mice. Together, these results underscore the impact of HFD feeding on the gut microbiota and inflammation and suggest the significance of diet-induced gut microbial dysbiosis and inflammation in promoting viral infection.

**IMPORTANCE:**

HFD induces gut microbial dysbiosis and inflammation and has been associated with many infections and disease progression; however, its impact on HIV-1 rectal transmission is largely unknown. Given the increasing threat of HIV-1 incidence in men who have sex with men (MSM), it has become crucial to comprehend the impact of factors associated with gut health, like HFD consumption, on host susceptibility to HIV-1 rectal transmission. This is particularly important since anal intercourse remains the primary mode of HIV transmission within the MSM group. In this study, utilizing our unique mouse model, featuring both the human immune system and gut microbiota, we showed that HFD feeding led to gut microbial dysbiosis, induced inflammation, and increased HIV-1 rectal transmission. Collectively, our study highlights the significant impact of HFD on gut microbiota and inflammation and suggests an HFD consumption as a potential risk factor for promoting HIV-1 rectal susceptibility.

## INTRODUCTION

New HIV-1 infections have been declining over the past two decades globally; however, their incidence in the men who have sex with men (MSM) population has continued to expand (1, 2). Since the onset of the HIV epidemic, MSM have been disproportionately affected by this virus. In the United States, 68% of HIV-1 new infections diagnosed in 2020 were from MSM (3). Recent studies have revealed unique compositional characteristics of the gut microbiome in MSM compared to that of men who have sex with women (MSW) regardless of HIV infection (4–6). Such alterations in the gut microbiome have been linked with increased immune activation and inflammatory markers, as well as enhanced susceptibility to HIV-1 transmission in MSM (7–9). Within the MSM population, these microbial variations, coupled with potential injury from receptive anal intercourse, may create a microenvironment that facilitates HIV-1 rectal transmission. In addition, compared to the proximal colonic section, the most distal section of the rectum is populated with greater concentrations of HIV target cells such as activated CD4+ T cells, macrophages, and dendritic cells, further fueling the risk of HIV transmission in MSM (10). Despite these links, further studies are needed to better understand how factors associated with gut health can disrupt gut homeostasis through microbial perturbation, metabolic inflammation, and immune modulation to influence rectal HIV-1 transmission.

Diets high in fat have profound effects on the gut microbiota and intestinal barrier functions. An HFD is well known to induce gut microbial dysbiosis that leads to increased microbial translocation and metabolic inflammation (11–13). Excessive dietary fats are increasingly linked to heightened intestinal permeability through multiple mechanisms, including changes in tight junction proteins, production of barrier-disrupting bile acids, stimulation of pro-inflammatory signaling cascades and cytokines, and induction of oxidative stress and apoptosis (14–16). In addition, studies in animal models have demonstrated that HFD feeding alters the phenotype of immune cells and enhances systemic immune activation and inflammation (17–20). Furthermore, HFD feeding has been linked to accelerated disease progression, including infection of Simian immunodeficiency virus (SIV), a close relative of HIV, in rhesus macaques (20, 21). Collectively, the combination of compromised intestinal barrier functions, gut microbial dysbiosis, and elevated levels of mucosal inflammation induced by an HFD may represent a risk factor for HIV-1 rectal transmission.

In this study, we assessed the impact of HFD feeding on the host’s susceptibility to HIV-1 rectal transmission utilizing our established double humanized bone-marrow, liver, thymus (dHu-BLT) mouse model featuring both an engrafted human immune system and a human fecal microbiota (22). We fed one group of dHu-BLT mice with an HFD to induce gut microbial dysbiosis and inflammation, while other dHu-BLT mice received a regular chow diet to serve as controls. We examined the effect of HFD feeding on both the gut microbiota and systemic immune activation and inflammation. Furthermore, we investigated the impact of HFD consumption on host susceptibility to HIV-1 rectal transmission. HFD feeding quickly changed the gut microbiome profile and increased both peripheral blood levels of inflammatory biomarkers and fecal calprotectin levels compared to controls. When repeatedly challenged with a low dose of HIV-1 *via* the rectal route, we found that HFD feeding exacerbated HIV-1 rectal transmission. This study provides useful insight into the impact of the HFD on the gut microbiota and systemic inflammation and suggests HFD consumption as a potential risk factor for HIV-1 rectal transmission and possibly a significant contributor to the increased incidence of HIV-1 infection in MSM.

## RESULTS

### Human donor fecal microbiota successfully engrafted in dHu-BLT mice

**Fig 1:**
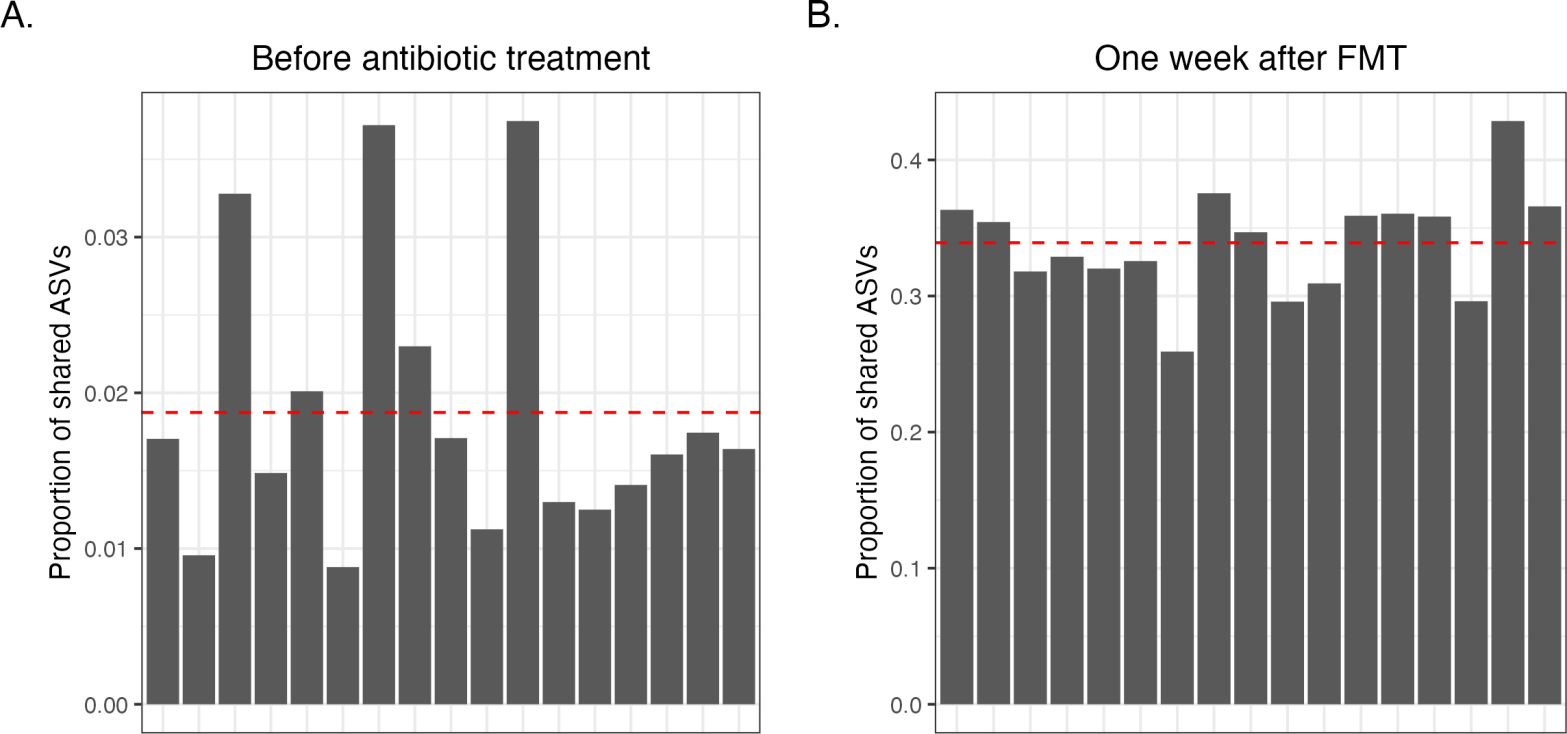
Successful engraftment of a portion of donor fecal microbiota into dHu-BLT mice. The proportion of shared ASVs of human donor fecal microbiota (Donor mix) with mice before antibiotic treatment (**A**) and 1 week after FMT (**B**). Each bar on the graph represents an individual mouse. The red dotted line represents the average shared ASVs.

Pre-treatment with a cocktail of broad-spectrum antibiotics has become a widely adopted practice for depleting the resident gut microbiota in mice prior to transplanting a donor microbiota (22–24). Treating Hu-BLT mice with ampicillin, metronidazole, neomycin, and vancomycin dramatically altered the gut microbial profiles of mice (Fig. S1), including a significant rise in the abundance of *Pseudomonadota* (formerly *Proteobacteria*). Following 14 days of antibiotic treatment, we engrafted an unbiased human donor sample (Donor mix) created by mixing an equal portion of three different healthy human donor samples (Fig. S2). We next compared the proportion of ASVs shared between the donor mix and the mouse gut microbiota before antibiotic treatment and 1 week after FMT. The average proportion of human donor mix ASVs shared with mice microbiotas was less than 2% before antibiotic treatment, while it was 33.91% after 1 week of FMT (Fig. 1). Furthermore, 23 ASVs from the donor mix were found in all dHu-BLT mice 1 week after FMT (Fig. S3; Table S10), suggesting successful engraftment of a portion of human fecal microbiota members in our double humanized mouse model.

### HFD induced gut microbial dysbiosis in dHu-BLT mice

**Fig 2:**
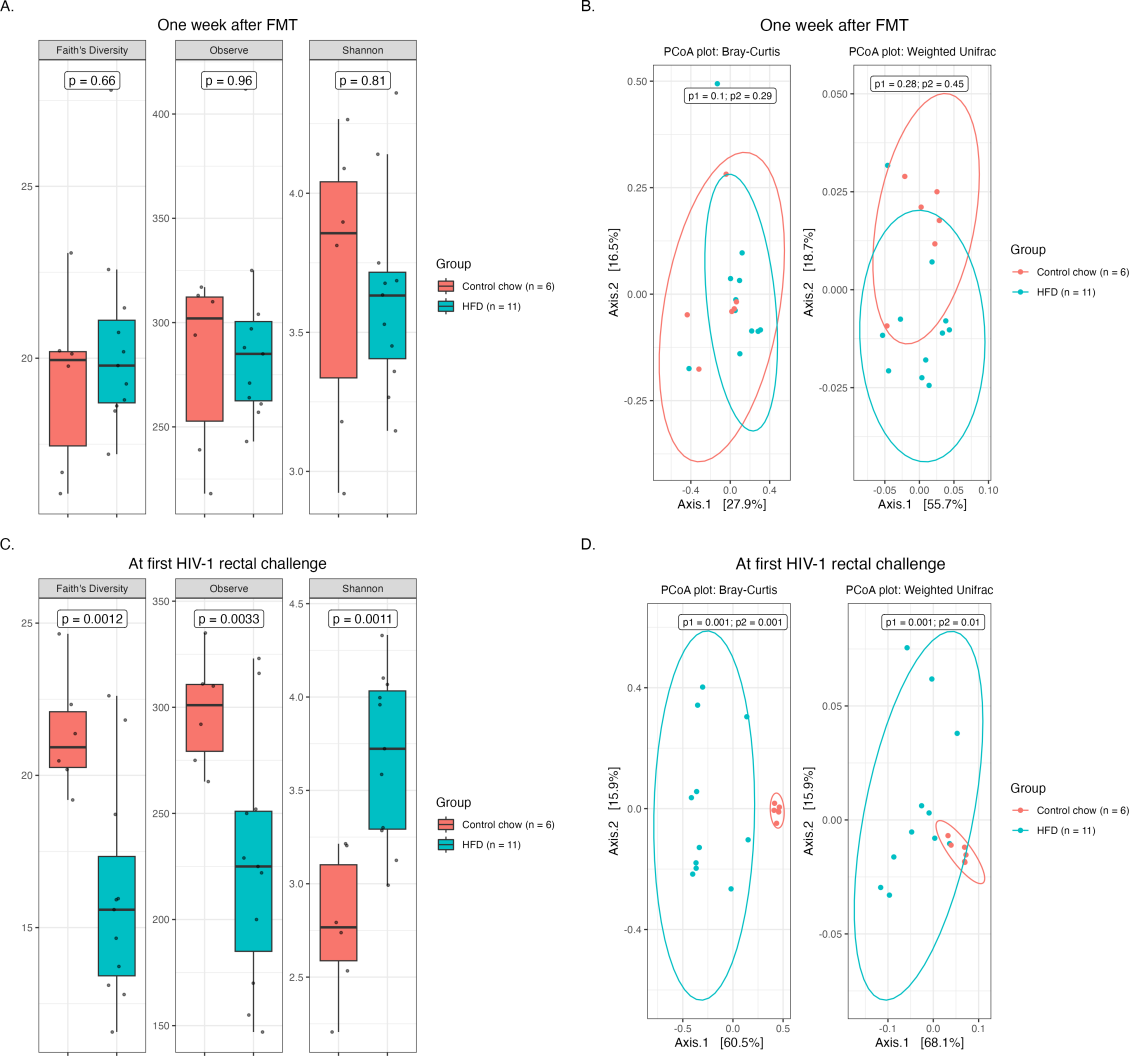
HFD feeding induced gut microbial dysbiosis in dHu-BLT mice. Alpha diversity (**A**) and beta diversity (**B**) metrics for mice at 1 week post-FMT (before HFD feeding); alpha diversity (**C**) and beta diversity (**D**) for mice at 3 weeks after HFD feeding was initiated in the HFD group (prior to the first HIV-1 rectal challenge). Each data point represents an individual mouse, p1 = Permutational Multivariate Analysis of Variance (PERMANOVA), and p2 = Permutational Analysis of Multivariate Dispersion (PERMDISP).

HFD is well documented to induce alterations in gut microbial composition and promote intestinal damage and inflammation (11–13, 16, 25–27). To assess the impact of an HFD on an engrafted human microbiota in our dHu-BLT mice, we first examined common metrics for alpha diversity (Observed ASVs, Shannon diversity index, and Faith’s phylogenetic diversity) and beta diversity (Bray-Curtis dissimilarity and weighted UniFrac). One-week post-fecal microbiota transplantation (FMT), no significant differences in alpha and beta diversity metrics were observed between the two sets of dHu-BLT mice under investigation (Fig. 2). However, within a week of feeding an HFD to one set of dHu-BLT mice, a noticeable shift in microbial diversity was evident (Fig. S4). After 3 weeks of HFD feeding (prior to the first HIV-1 rectal challenge), all evaluated alpha metrics were significantly different between HFD-fed mice and controls. Both Faith’s phylogenetic diversity and the number of observed ASVs were significantly lower for HFD mice compared to controls, while the Shannon diversity index was significantly higher. Also, in Bray-Curtis dissimilarity and weighted UniFrac beta diversity metrics, both diet groups clustered separately (Fig. 2).

**Fig 3:**
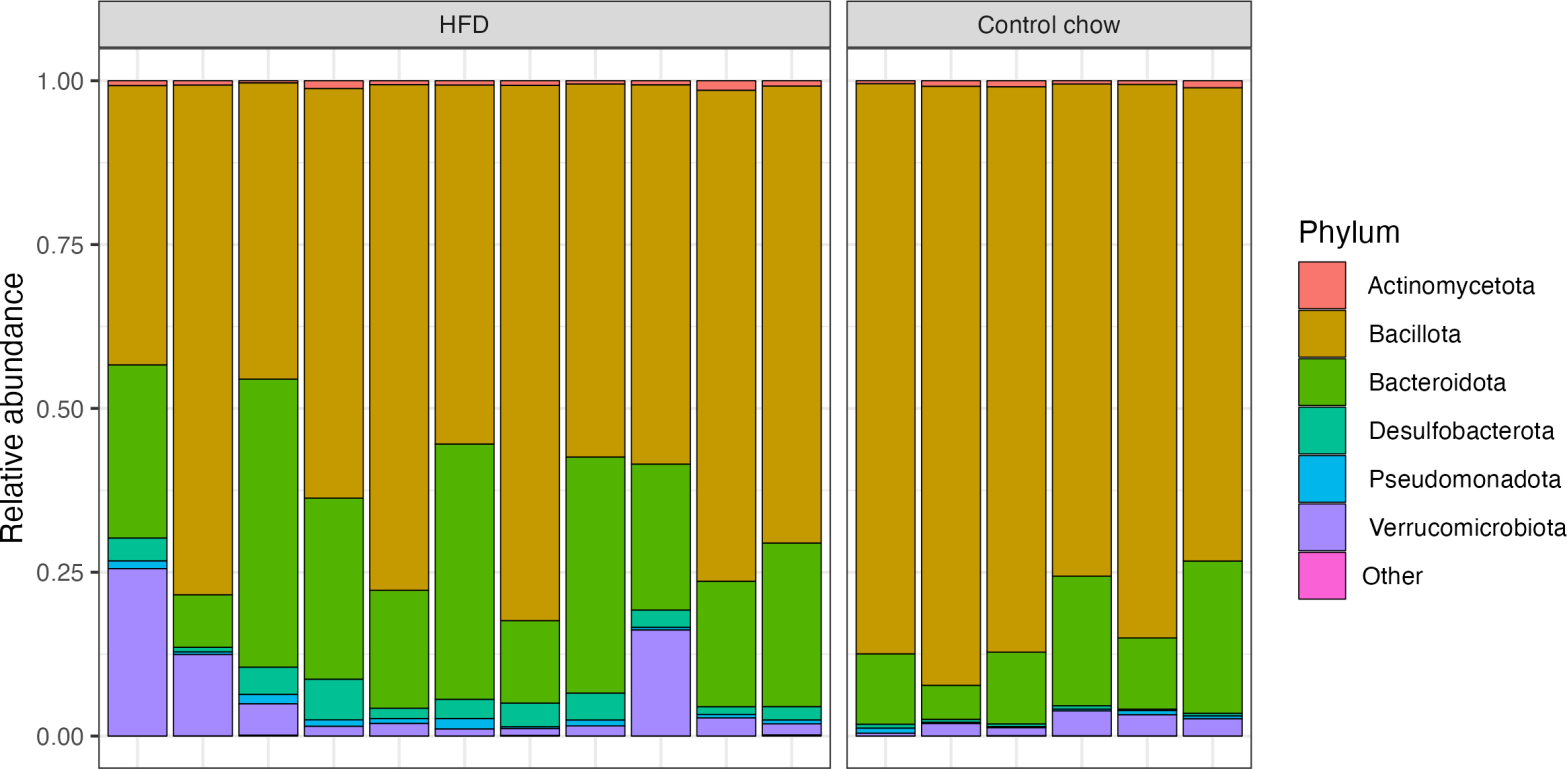
Distinct microbial composition differences at the phylum level in mice fed HFD versus control chow. The figure illustrates the relative abundance of different phyla in each mouse at 3 weeks post-HFD feeding to the HFD group (prior to the first HIV-1 rectal challenge). Each bar on the graph represents an individual mouse. Phylum <0.005 abundance is grouped as "Other."

**Fig 4:**
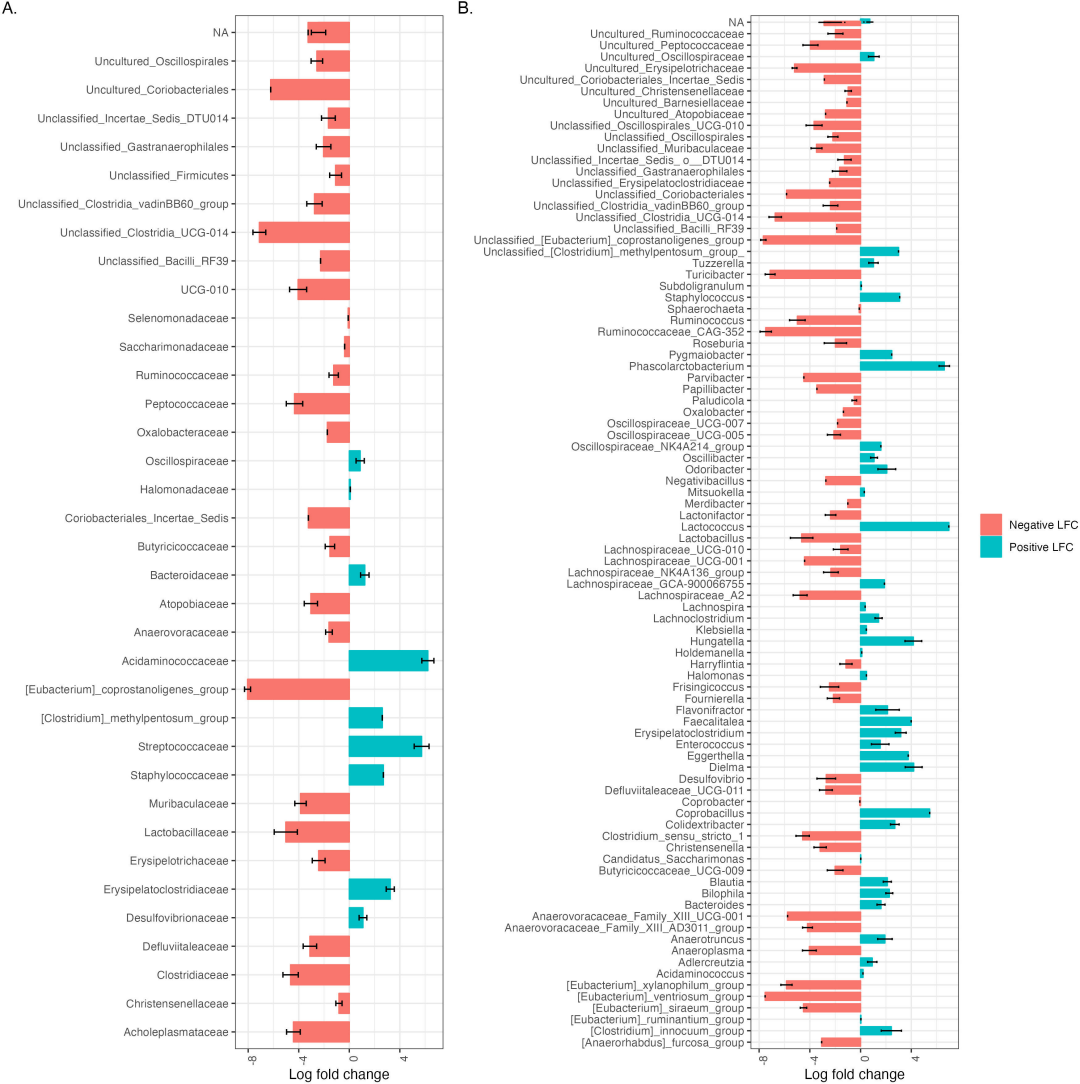
The HFD-fed mice exhibited a decrease in absolute abundance for the majority of differentially abundant taxa. Waterfall plot of log fold change (natural log) of absolute abundance of differentially abundant taxa (A) at the family level and (B) at the genus level. NA represents unassigned taxa at respective levels of taxonomic resolution. Unclassified taxa with the respective level of taxonomic resolution were renamed to the nearest classified taxa. Only significant log-fold changes (with fdr *P*-value < 0.05) from the result of ANCOM-BC analysis are shown.

We further investigated differences in the microbial compositions of the individual mice from each diet group 3 weeks after initiating HFD feeding. First, we measured the relative abundance of different phyla in each mouse. The majority of HFD-fed mice had a relatively higher abundance of *Bacteroidota*, *Desulfobacterota*, *Pseudomonadota* (formerly *Proteobacteria*), and *Verrucomicrobiota* compared to controls (Fig. 3). We then searched for differentially abundant taxa using Analysis of Composition of Microbiomes with Bias Correction (ANCOM-BC). Four phyla— *Cyanobacteriota*, *Desulfobacterota*, *Bacillota* (formerly *Firmicutes*), and *Patescibacteria—*were differentially abundant between dietary treatments (Fig. S5). At the family-level resolution, 35 taxa and 89 taxa at the genus-level resolution were identified as differentially abundant between groups (Fig. 4). The highest log_2_ fold change at the family level was observed in the increase in *Acidaminococcaceae* and decrease in *[Eubacterium]_coprostanoligenes_group*. Notably, common pathobionts such as *Staphylococcaceae* were only present in the HFD-fed mice, and beneficial taxa like *Lactobacillaceae* were observed in reduced abundance as compared to the control mice. In addition, *Uncultured_Coriobacteriales*, *Coriobacteriales_Incertae_Sedis*, *Unclassified_Bacilli_RF39*, *Oxalobacteraceae*, *Saccharimonadaceae*, and *Selenomonadaceae* were absent in the HFD group while *Halomonadaceae* and *[Clostridium]_methylpentosum_group* were absent in the controls. Similar to family level analysis, the majority of genera were reduced in abundance in HFD-fed mice as compared to controls, including *unclassified_[Eubacterium]_coprostanoligenes group* and *[Eubacterium]_ventriosum_group* and *Ruminococcaceae_CAG-352 group*. There were significant increases in the abundance of *Lactococcus* [detected only in HFD-fed mice; likely a dietary contribution (28)], *Phascolarctobacterium,* and *Coprobacillus*. Furthermore, 135 taxa with species-level resolution were found to be differentially abundant between diet groups (Table S4). Together, these data demonstrate significant alterations in the engrafted human microbiota upon HFD intervention.

### HFD feeding elevated inflammatory markers and fecal calprotectin levels in dHu-BLT mice

**Fig 5:**
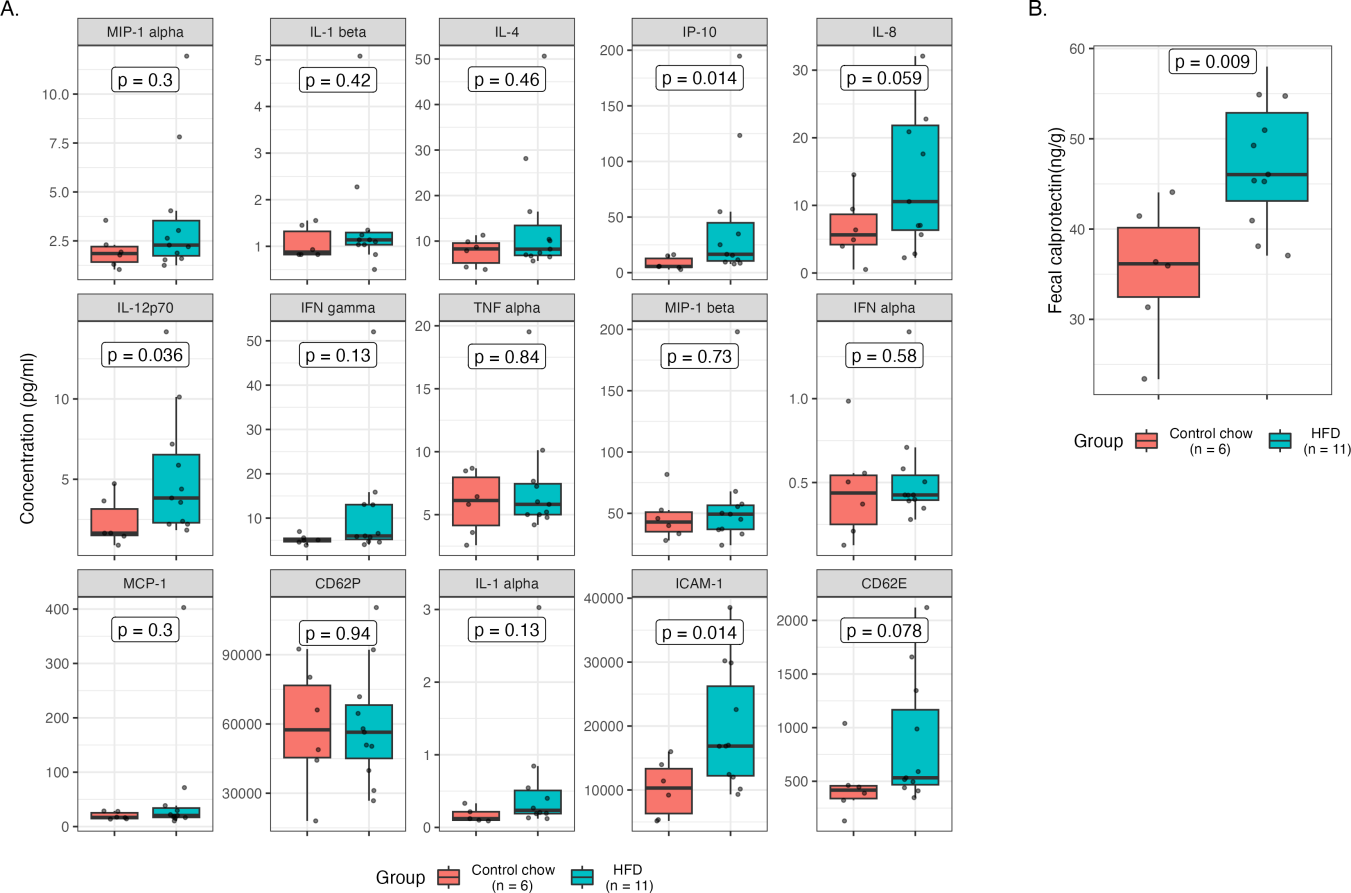
HFD feeding elevated plasma inflammatory markers and fecal calprotectin levels in dHu-BLT mice. (**A**) Inflammatory biomarkers measured in plasma samples prior to the first HIV-1 rectal challenge. Each data point represents the mean concentration (pg/mL) of respective inflammatory biomarkers measured in an individual dHu-BLT mouse’s duplicate plasma sample. (**B**) Fecal calprotectin levels prior to the first HIV-1 rectal challenge. Each data point represents the mean fecal calprotectin concentration (ng/g) measured in an individual dHu-BLT mouse’s duplicate fecal sample. A *P*-value < 0.05 is considered significant.

Inflammation and immune activation at the mucosal portal of virus entry have been associated with increased susceptibility to HIV infection and virus shedding (29–31). Inflammation can compromise mucosal epithelial barrier integrity and function and recruit HIV-1 target cells, thereby creating a favorable niche for virus transmission and promoting productive infection (32, 33). To understand the role of HFD on inflammation and immune activation and, in turn, on the risk of HIV-1 rectal transmission, we measured multiple inflammatory markers and activated T-cell populations. Of the 20 different plasma inflammatory mediators measured (Table S5), 15 were detected in the majority of the samples. Levels of IL-12p70, IP-10, and ICAM-1 were significantly different between the diet groups (Fig. 5A). In addition, the level of human fecal calprotectin, an important marker for inflammation in the gastrointestinal tract, was significantly higher in HFD-fed mice versus controls (Fig. 5B). However, the population of activated T cells measured in peripheral blood was not statistically different between dietary treatments (Fig. S6). Together, these results suggest ongoing inflammation both locally and systemically in HFD-fed mice.

### Inflammatory markers (IL-12p70 and ICAM-1) altered by HFD feeding were negatively correlated with alpha diversity metrics

**Fig 6:**
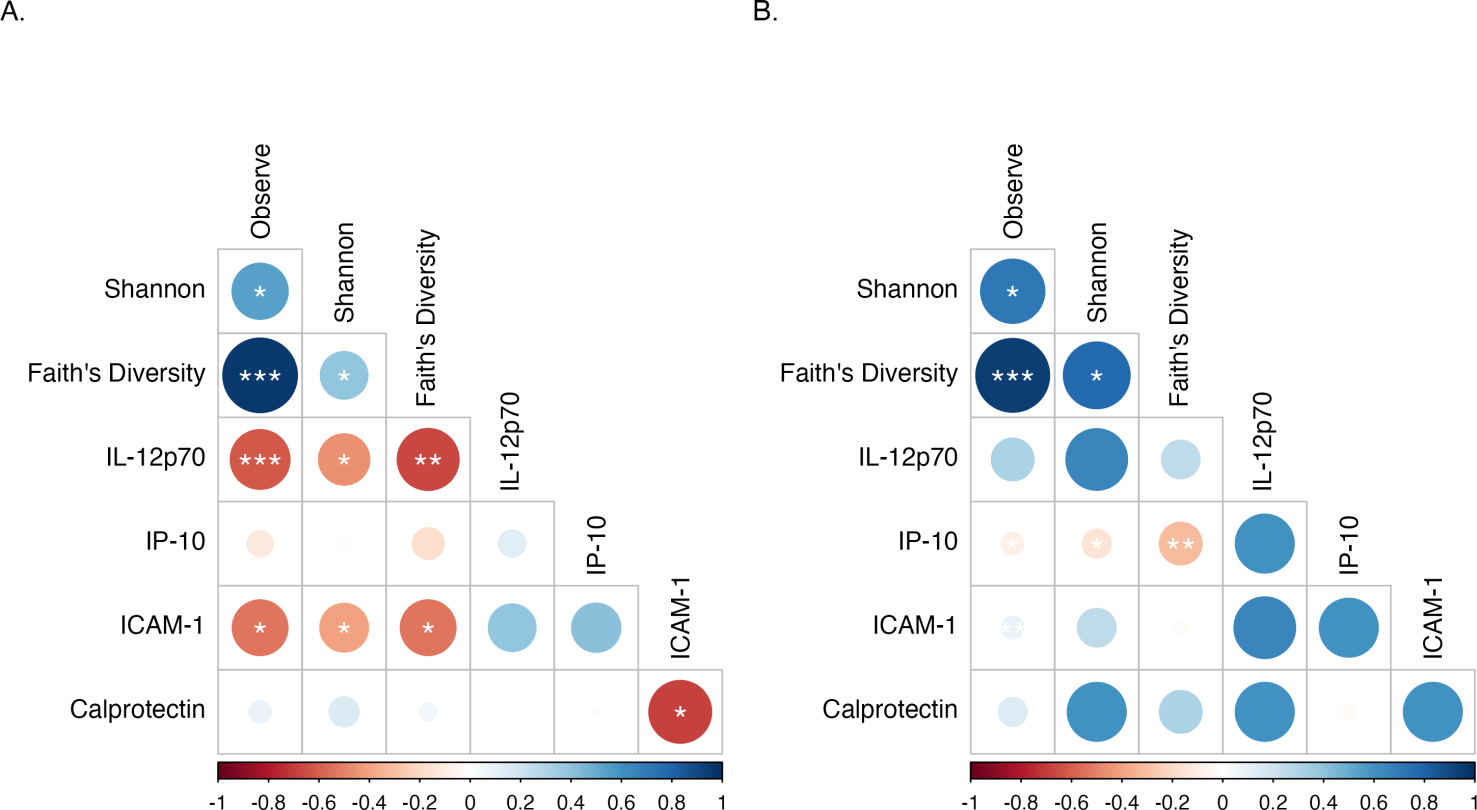
Inflammatory markers (IL-12p70 and ICAM-1) altered by HFD feeding were negatively correlated with alpha diversity metrics. Correlation of alpha diversity indices and significantly different inflammatory markers in HFD group (**A**) and control chow group (**B**). Asterisks indicate significant correlation determined by Spearman’s test; *, **, and *** indicate significant differences with *P* < 0.05, *P* < 0.01, and *P* < 0.001, respectively. Circle sizes are proportional to the strength of correlation.

The gut microbiota influences a variety of host physiological processes, and changes in gut microbial composition, particularly reduced alpha diversity, have been linked to markers of metabolic dysfunction (34, 35). We, therefore, sought to test for correlations of microbial diversity with inflammatory markers observed to be significantly altered by HFD feeding. Notably, all the alpha diversity indices evaluated in our study were negatively correlated with the level of IL-12p70 (Observed ASVs: *P* < 0.001, r = −0.62; Shannon: *P* < 0.05, r = −0.45; Faith’s diversity: *P* < 0.01, r = −0.66) and ICAM-1 (Observed ASVs: *P* < 0.05, r = −0.54; Shannon: *P* < 0.05, r = −0.41; Faith’s diversity: *P* < 0.05, r = −0.54) in mice-fed HFD (Fig. 6A). By contrast, only IP-10 in the control mice (Fig. 6B) was negatively correlated with the alpha diversity matrices evaluated (Observed ASVs: *P* < 0.05, r = −0.09; Shannon: *P* < 0.05, r = −0.14; Faith’s diversity: *P* < 0.01, r = −0.31) in our study. Collectively, these associations suggest that a reduction in microbial alpha diversity could potentially be a contributing factor to the heightened inflammation observed in HFD-fed mice.

### HFD feeding increases the risk of HIV-1 rectal transmission

**Fig 7:**
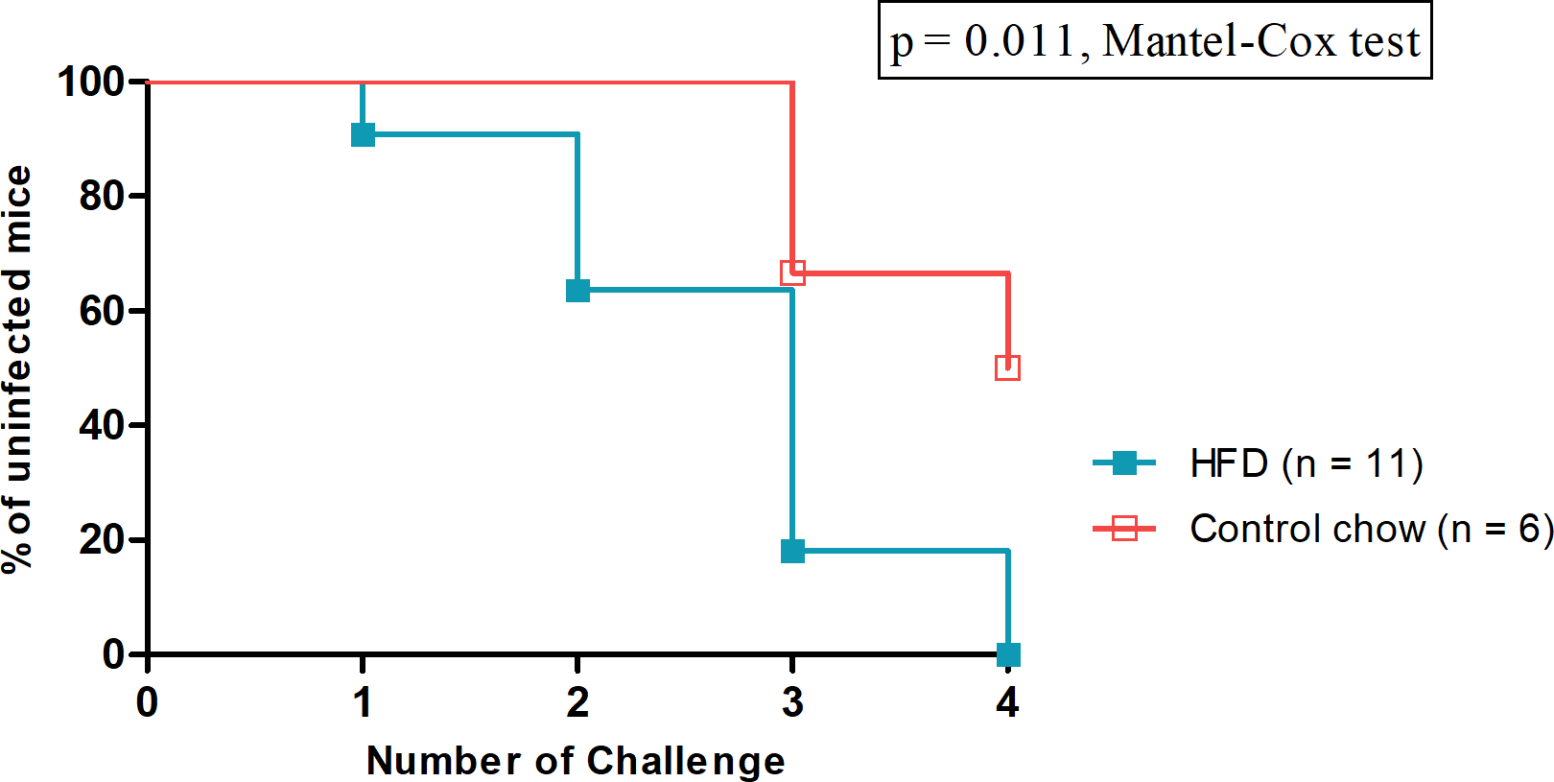
HFD feeding enhanced HIV-1 rectal susceptibility. Kaplan-Meier plot of the percentage of uninfected mice versus the number of challenges is displayed in the graph. Statistical comparison is performed using the Mantel-Cox test. A *P*-value < 0.05 is considered significant.

HFD feeding has been linked to increased host susceptibility to bacterial enteric infection (36, 37) and disease progression of SIV-infected macaques (20, 21). To evaluate the impact of HFD feeding on HIV-1 rectal infection, we rectally challenged both control chow and HFD-fed dHu-BLT mice once every 2 weeks with a repeated low dose of HIV-1. The cumulative proportion of mice that remained uninfected over four low doses of HIV-1 rectal challenges is shown in Fig. 7. All HFD-fed mice (11 out of 11) were infected after the fourth challenge (Table S9). By contrast, only 50% of the control mice (3 of 6 mice) were infected during this period (Table S9). The difference in the risk of HIV-1 rectal transmission between the two diet groups was statistically significant (*P* = 0.011, Mantel-Cox test). These results demonstrate that HFD feeding is associated with an increased risk of HIV-1 rectal transmission.

## DISCUSSION

Several studies have shown that the risk of HIV transmission through receptive anal intercourse is higher than the risk associated with other sexual activities, such as vaginal intercourse or oral sex (38). With the rising incidence of HIV-1 infection in MSM, it is plausible that factors associated with the gastrointestinal tract may contribute to the increasing HIV-1 prevalence in this population. In the female genital tract, bacterial vaginosis and associated dysbiosis of the vaginal microbiome are considered major contributors to vaginal inflammation and increased risk of HIV-1 vaginal acquisition (39–41). However, the impact of gut microbial alteration and inflammation in HIV-1 rectal transmission has not been well established. Anatomically, compared to the vaginal mucosa, which consists of multilayered stratified squamous epithelium, rectal tissue is a single layer of columnar epithelial cells, thus making them more vulnerable to infection through mechanical trauma during sexual intercourse (38). In addition, there is growing evidence of unique compositional microbiome characteristics in MSM compared to MSW (4–6), where changes in the gut microbiome are associated with an increased risk of HIV-1 susceptibility (8). Furthermore, there is evidence of elevated expression of the HIV-1 co-receptor CCR5 in CD4+ T cells in rectosigmoid colonic biopsies from MSM versus MSW (42) and fecal bacterial communities from MSM inducing higher frequencies of CCR5+ CD4+ T cells during *in vitro* assays than those from MSW (43). Altogether, findings from these reports suggest potential connections among anatomical features of the lower gastrointestinal tract, gut microbial composition, and HIV-1 rectal susceptibility in MSM. Considering the increasing threat of HIV-1 incidence in MSM (1–3), where anal intercourse remains the primary mode of HIV transmission (44), it is crucial to explore the risk factors linked to gut health and their impact on HIV-1 rectal transmission.

HFD is well known to affect multiple aspects of gut health, ranging from the structural integrity of the gut to the composition of resident microbiota (11–14, 25–27, 45). Numerous diseases and inflammatory conditions have been associated with HFD consumption. In humans, diets rich in fat sources, a typical feature of Western diets, are associated with obesity, nonalcoholic fatty liver disease (NAFLD), type 2 diabetes mellitus, cardiovascular diseases, and cerebrovascular pathology, including cognitive impairment and emotional disorders (46, 47). Studies in rhesus macaques have demonstrated that a diet rich in fat accelerated SIV disease progression (20, 21). However, in terms of HIV-1, particularly in rectal transmission, the impact of an HFD and its associated microbial dysbiosis and inflammation is largely unknown. The challenge with such studies lies in the limited availability of suitable animal models where the combination of the human immune system, the human gut microbiota, and HIV-1 can be studied simultaneously with dietary interventions. Thus, the causal role of diet-induced inflammation and gut microbial dysbiosis in HIV-1 rectal transmission remains largely undefined. This study sought to close this knowledge gap by addressing the question of whether inflammation and microbial perturbation induced due to HFD feeding enhances the risk of HIV-1 rectal transmission by utilizing our established novel dHu-BLT mouse model featuring a stable human-like gut microbiome and human immune system (22).

The dHu-BLT mouse model provides a unique opportunity to study the relationships among a functional human immune system, a human-like microbiome, and HIV-1 infection. The BLT method of humanization enables robust engraftment of the human immune system within the gastrointestinal tract of BLT mice and has been extensively used for HIV rectal transmission studies (48). The human immune reconstitution of the dHu-BLT mice used in the current study was similar to that previously reported for BLT mice (49, 50). In addition, one-third of the human donor microbiota taxa were detected 1 week after FMT in the dHu-BLT mice used in the current study, suggesting the successful engraftment of a portion of the human gut microbiota as we reported previously (22, 51). To assess the impact of HFD feeding on the host’s susceptibility to HIV-1 rectal transmission, we fed one group of mice with an HFD, which is well documented to induce notable changes in gut microbial composition and inflammation. After 3 weeks of HFD feeding, all measures of alpha diversity evaluated were significantly different between the diet groups. HFD is reported to decrease common matrices for alpha diversity (28, 52), but in our study, the Shannon diversity index was higher in the HFD group compared to the controls. The difference between our observation versus those of others could be due to the mouse strain and model used in our study. A similar effect of increased Shannon diversity in different mouse strains fed with an HFD has been previously reported by others (27, 45). Another study has also reported an increase in the Shannon diversity index in mice fed with an HFD over time (26), which is consistent with our study in which the Shannon diversity index increased considerably during the time between the human microbiota transplant and 3 weeks of HFD feeding (Fig. 2A and C). We further investigated the differentially abundant taxa between the diet treatments prior to starting the HIV-1 rectal challenge. At the family level, *Desulfobacterota* and *Bacillota* (formerly *Firmicutes*) were differentially abundant in ANCOM-BC analysis (Fig. S5). *Desulfobacterota,* a sulfate-reducing species, is associated with increased immune responses, gut inflammation, and higher fecal IgA concentrations (53, 54), while the reduction of *Bacillota* (formerly *Firmicutes*) has been linked to increased systemic inflammation (55) and human inflammatory diseases like inflammatory bowel disease (IBD) (56). In addition, common pathobionts like *Staphylococcaceae* were only detected in mice fed an HFD prior to HIV-1 rectal challenge. Pathobionts such as *Staphylococcaceae* are known to prosper in an inflamed gut and support inflammation (57). By contrast, beneficial taxa such as *Lactobacillaceae,* which have been suggested to prevent inflammation (58, 59), were decreased in abundance during HFD feeding compared to the controls. Furthermore, some beneficial bacterial species suggested to play a protective role against inflammation such as *Lactobacillus johnsonii* (60)*, Alistipes finegoldii* (61)*, Bacteroides xylanisolvens* (62), *Bacteroides uniformis* (63), and *Oxalobacter formigenes* (64) were found in reduced abundance in the HFD group while opportunistic like *Bacteroides nordii* (65) were in increased abundance (Table S4) prior to HIV-1 rectal challenge. These bacteria are known to modulate the host physiology *via* their metabolites or by influencing inflammatory signaling pathways. For instance, *Lactobacillus johnsonii* L531 inhibits the TLR4/NF-κB/NLRP3 inflammasome signaling pathway to alleviate *S. typhimurium*-induced tight junction damage (60). *Alistipes finegoldii* mediates protection from dextran sodium sulfate (DSS)-induced colitis by restoring intestinal barrier function through the enhanced colonic expression of intestinal IL-22 and *Reg3γ* (61). Likewise, a recent investigation has demonstrated the ability of *Bacteroides xylanisolvens* AY11-1 to generate oligosaccharides and beneficial short-chain fatty acids through the breakdown of alginate and its derivatives (62). Moreover, this study also revealed that *B. xylanisolvens* attenuated mucosal damage in mice with DSS colitis (62). Consequently, the increase in these potentially pro-inflammatory bacteria and a concomitant loss of beneficial organisms may have contributed to the inflammation detected in our HFD-fed mice due to altered production of gut microbial metabolites or their influence on the host gene expression.

Consumption of an HFD has been linked to increased inflammatory markers and immune modulation (12, 17–21, 25, 46). In addition, several studies have found that elevated levels of inflammatory markers and immune activation are associated with increased HIV-1 susceptibility and infectiousness (9, 29, 30, 66, 67). In our study, we observed that IL-12p70, IP-10, and ICAM-1 were significantly higher in the HFD-fed mice compared to controls (Fig. 5A). IL-12p70 is an important immunoregulatory cytokine, primarily produced by professional antigen-presenting cells (APC) that induce Th1 cells differentiation as well as activation of natural killer cells (68). The microbial dysbiosis and immune activation prompted by our HFD could be a key stimulant of elevated IL12p70 in the HFD-fed mice as microbial products and activated T cells and APC interaction are known to directly induce IL-12 secretion (69). Furthermore, IL12p70 has been linked to tissue injury and is a major factor in the pathogenesis of Crohn’s disease (70, 71). IP-10 was another inflammatory modulator found to be significantly different between the diet groups. Like IL12p70, higher expression of IP-10 has been linked to chronic intestinal inflammation and tissue damage (72). This chemokine mediates apoptosis and facilitates bacterial and viral infection, including HIV-1 (73, 74). In addition, several studies have correlated elevated levels of IP-10 with an increased HIV-1 acquisition risk and disease pathogenesis (29, 66, 67, 75, 76). Interestingly, in our study, the HFD-fed mice were the most susceptible to low-dose HIV-1 rectal challenge and also had significantly higher levels of IP-10 compared to controls. We also observed significantly higher expression of ICAM-1, a master regulator of cellular responses in inflammation, in our HFD-fed mice. Similar results of elevated ICAM-1 expression in mice fed with an HFD have also been reported in other studies (25, 77). Like IP-10, elevated levels of ICAM have been associated with enhancing HIV-1 disease progression (78, 79). In addition, the levels of detection in plasma were out of range (<OOR) for the pro-inflammatory cytokine IL-6 in chow-fed dHu-BLT mice; however, IL-6 was detected in some of the HFD-fed mice, suggesting an ongoing inflammatory response in these animals (Table S5). Also, the cytokines IL-10 and IL-13 were detected in the majority of HFD-fed mice compared to control animals, which could be the result of a suppressive immune response initiated due to elevated inflammation from the HFD feeding. Calprotectin accounts for the majority of the cytosolic proteins in neutrophils, and its presence in feces indicates neutrophilic infiltration into the gut lumen due to an inflammatory process or infection (80). Compared to chow-fed mice, the level of human fecal calprotectin was significantly higher in the HFD-fed mice (Fig. 5B), suggesting notable ongoing inflammatory activity during HFD feeding. In recent studies, it has been demonstrated that HFD feeding alters the phenotype of immune cells. Specifically, the studies revealed an increase in the population of activated T cells, polarization of macrophages, and proliferation and activation of invasive Ly6C^high^ monocytes in HFD-fed mice (17, 18). However, in our current study, we did not observe any statistically significant difference in the T-cell activation between diet treatments prior to the HIV-1 rectal challenge (Fig. S6). This result could be due to the duration of HFD feeding, which is considerably shorter than what has been published by others (17, 18).

Several studies have reported an association between altered microbiomes and inflammation (81, 82). Dysregulated microbiomes can impair epithelial barrier function and gut mucosal homeostasis (83, 84). In addition, the translocation of microbial products as a result of compromised intestinal integrity has the potential to drive systemic inflammation and disease progression (83, 84). Here, we further investigated the correlation between microbiome diversity indices with inflammatory markers that were significantly different between diet treatments. We observed that plasma levels of the inflammatory mediators IL-12p70 and ICAM-1 were negatively correlated with all the alpha diversity indices evaluated for HFD-fed mice (Fig. 5A). Species diversity plays an important role in the maintenance of intestinal homeostasis and increases in diversity have been generally associated with health (85) while reductions in diversity are often viewed as potential initiators of inflammatory reactions (86). Increased levels of inflammatory mediators observed in our HFD mice could have resulted from the loss of potentially beneficial bacteria responsible for maintaining the intestinal ecosystem or may have been due to the increased translocation of microbial products due to impaired barrier function.

Finally, our low-dose HIV-1 rectal challenge study showed that mice fed an HFD are more susceptible to an HIV-1 rectal challenge (Fig. 7). By the fourth rectal challenge, all the HFD-fed dHu-BLT mice were infected compared to only 50% of those fed a control chow diet. This result demonstrates that HFD feeding enhances HIV-1 rectal transmission. In our study, it is plausible that HFD feeding initiated some degree of pathophysiological and immunological changes to the gastrointestinal tract of dHu-BLT mice. In turn, these effects may have increased local inflammation and mucosal membrane permeability, thus leading to microbial translocation and systemic inflammation. In addition, the number of activated CD4+ T cells at the site of inflammation may have also been increased, thereby providing additional target cells to facilitate HIV-1 infection. Altogether, these various scenarios, coupled with diet-induced microbial perturbations, may have created a favorable microenvironment for successful HIV-1 transmission.

Our study has several important implications regarding the impact of HFD feeding on susceptibility to HIV-1 rectal transmission. In our current study, we extensively characterized the effects of consuming an HFD on gut microbial composition and inflammatory mediators prior to the HIV-1 rectal challenge and assessed its impact on the host’s susceptibility to HIV-1 rectal transmission. Such studies in human subjects are difficult to perform due to ethical constraints and many confounding factors such as human behavior, socioeconomic factors, variation of dietary intake, medication, and host genetics. However, in the current study, we circumvented those issues and assessed the effect of an HFD on HIV-1 rectal susceptibility in a strictly controlled environment. In addition, this study not only recapitulated the profound effects of an HFD on the gut microbiota and inflammation but also linked diet-induced gut microbial dysbiosis and inflammation in the context of viral infection. Moreover, the HFD used in our study contained high levels of fat and sucrose but had low fiber content. These features are typical of the Western diet, which may be a factor contributing to HIV-1 infection among MSM from developed nations. This diet formulation is particularly relevant in light of the increased incidence of HIV-1 among MSM from developed countries (western and central Europe and North America) (1). Furthermore, our comprehensive characterization of inflammatory mediators and T-cell activation in plasma adds additional valuable information to the current understanding of systemic immune activation and inflammation due to HFD feeding.

Although our work provides useful insight into the influence of HFD feeding on the human gut microbiota, inflammation, and HIV-1 rectal transmission, it is crucial to acknowledge some inherent limitations associated with our animal model. These limitations include the challenge of achieving the complete engraftment of the human donor microbiota in the murine gut and the anatomical dissimilarities of mice compared to the human lower gastrointestinal tract. Moreover, our assessment of gut inflammation over time relied solely on fecal calprotectin levels. Yet, the characterization of inflammatory mediators and activated immune cells at the local gut mucosa prior to the HIV-1 rectal challenge could have provided additional insight into the ongoing pathophysiological conditions at the site of transmission. Despite these limitations, our study demonstrates that consuming an HFD alters the gut microbiota, enhances inflammation, and increases HIV-1 rectal transmission in well-controlled animal model. In a broader context, this study highlights the ramifications of an altered gut microbiota and inflammation due to an HFD on the establishment of a viral infection. Importantly, our work supports further study on using either an FMT or dietary intervention to correct gut microbial dysbiosis as a prevention strategy to limit HIV-1 rectal transmission.

## MATERIALS AND METHODS

### Generation of hu-BLT mice

Hu-BLT mice were generated as we previously reported (49, 87). Briefly, NSG (NOD.*Cg-Prkdc^scid^Il2rg^tm1Wjl^*/SzJ; Cat# 005557; Jackson Laboratory, Bar Harbor, ME) female mice of 6–8 weeks were housed at UNL animal facility under specific-pathogen-free conditions. On the day of surgery, mice were subjected to sublethal irradiation at the dose of 12 cGy per gram of body weight using the RS200 X-ray irradiator (RAD Source Technologies, Inc., GA). Mice were then surgically implanted with a piece of human fetal thymic tissue fragments sandwiched between two pieces of human fetal liver tissue within the left renal capsule, followed by intravenous injection of 1.5–5 × 10^5^ CD34^+^ hematopoietic stem cells isolated from human fetal liver within 6 hours of surgery. Human fetal thymus and liver tissue were procured from Advanced Bioscience Resources (Alameda, CA). Ten weeks after surgery, human immune reconstitution was measured using flow cytometry as described below.

### Antibiotic treatment and human fecal microbiota transplant

Hu-BLT mice with similar human immune reconstitution were used to generate dHu-BLT mice following our previously reported method (22). Briefly, a broad-spectrum antibiotic cocktail of metronidazole (1 g/L), neomycin (1 g/L), vancomycin (0.5 g/L), and ampicillin (1 g/L) along with grape-flavored Kool-Aid was freshly prepared daily and given to hu-BLT mice for 14 days. During antibiotic treatment, cages were changed daily to limit re-inoculation of pre-existing bacteria in the mice due to coprophagic behavior. After 14 days of broad-spectrum antibiotic treatment, mice were given non-acidified autoclaved deionized water for 24 hours, followed by two oral gavage of 200 µL of human fecal microbiota mixture (Donor mix) at 24-hour intervals using an animal feeding needle (Cat# 9928B; Cadence science). The human fecal microbiota mixture (Donor mix) was prepared by mixing an equal portion of each donor sample from three different healthy human donors (two males and one female, aged 26–29 years; OpenBiome, Massachusetts, US) in an anaerobic chamber.

### Human immune reconstitution and immunophenotyping

Human immune reconstitution and immunophenotypes of hu-BLT and dHu-BLT mice were measured in peripheral blood using a fluorescence-activated cell sorter (FACS) Aria II flow cytometer (BD Biosciences, San Jose, CA) and antibodies against mCD45-APC, hCD45-FITC, hCD19-PE/Cy5, hCD3-PE, hCD4-Alexa 700, hCD8a-APC-Cy7, hCD69-BV 785, hCD38-BV 421, and hHLA-DR-PE/Cy7 (catalog numbers 103111, 304006, 302210, 300408, 300526, 301016, 310932, 356618, and 307616 respectively; Biolegend, San Diego, CA). Raw data were analyzed with FlowJo (version 10.8.1; FlowJo LLC, Ashland, OR). All hu-BLT mice used in this study had >50% of human CD45+ reconstitution in the mouse peripheral blood lymphocytes analyzed (Table S8).

### Experimental approach

**Fig 8:**
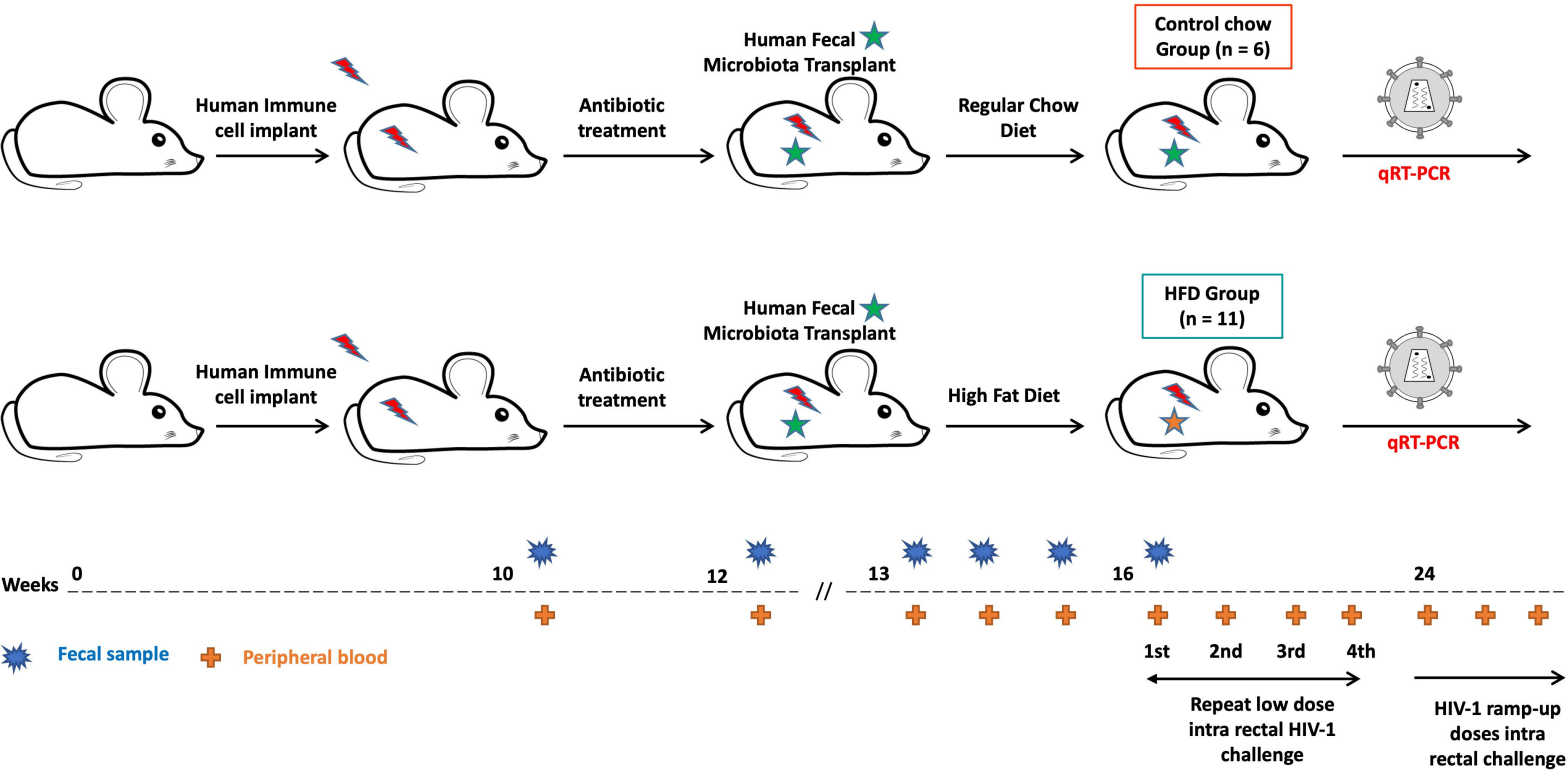
Two groups of dHu-BLT mice were generated by surgically implanting a human immune system followed by a human fecal microbiota transplant. One week post-FMT, one group of dHu-BLT was continued with a regular chow diet, and the second group of dHu-BLT mice was fed with HFD to create a control chow group and an HFD group, respectively. Three weeks after initiating HFD feeding to the HFD group, mice from both groups were challenged with a low dose of HIV-1 *via* the rectal route until all the mice in the HFD group were infected, followed by HIV-1 ramp-up doses challenged to uninfected mice. Fecal samples and peripheral blood were collected at different time points for microbial and inflammatory biomarkers analyses.

The dHu-BLT mice generated as described above were divided into two groups. Both groups of mice were initially fed regular mouse chow (Teklad 2916; Inotiv, Madison, WI). One-week post-human fecal microbiota transplant, one group of mice (HFD group, *n* = 11) was switched to a diet containing 45% kcal from fat (D12451i; Research Diets, New Brunswick, NJ) to induce gut microbial dysbiosis while another group (Control chow, *n* = 6) continued receiving regular mouse chow. Three weeks after the HFD feeding was initiated, all dHu-BLT mice were challenged intrarectally with a low dose of transmitted/founder HIV-1 SUMA (1 × 10^4^ TCID_50_ in 20 μL) once every 2 weeks under isoflurane anesthesia as reported previously (88). Infection was evaluated 2 weeks after each challenge *via* quantitative reverse transcription PCR (qRT-PCR). Once all HFD-fed mice were infected, uninfected mice in the control group were challenged with HIV-1 ramp-up doses (10-fold increase of TCID_50_ in every challenge) every 2 weeks until all mice were infected. Fecal samples and peripheral blood were collected at multiple time points to evaluate gut microbiome profiles and systemic immune activation, respectively (Fig. 8).

### Quantification of plasma viral loads

Plasma viral loads were measured using qRT-PCR as described previously (89) 2 weeks after each HIV-1 rectal challenge. Briefly, a QIAamp Viral RNA mini kit (Qiagen, Cat# 52906) was used to extract viral RNA from the mouse plasma and HIV-1 analytical standards in the AcroMetrix HIV-1 panel (ThermoFisher Scientific, Cat# 950470) following the manufacturer’s protocol. Viral RNA was then quantified using a C1000 Thermal Cycler and the CFX96 Real-time system (Bio-Rad). The reaction mixture consisted of 5 µL extracted viral RNA, TaqMan Fast Virus 1-Step master mix (ThermoFisher Scientific, Cat# 4444432), forward primer (5′-GCCTCAATAAAGCTTGCCTTGA-3′), reverse primer (5’- GGGCGCCACTGCTAGAGA-3), probe (/56-FAM/CCAGAGTCA/ZEN/CACAACAGACGGGCACA/3IABkFQ/), and nuclease-free water to achieve a total volume of 20 µL.

### Fecal sample collection and DNA extraction

Freshly collected mouse fecal samples were stored in 1.5 mL autoclaved Eppendorf tubes at −80°C until DNA extraction. Fecal DNA was extracted following the method described by Martinez et al. (90). Briefly, fecal samples were washed three times with ice-cold phosphate-buffered saline (Cat# SH30256.02, Hyclone) followed by physical lysis of cells using bead beater (Mini-Beadbeater-16, Biospec) in the presence of lysis buffer, 0.1 mm zirconia/silica beads (Biospecs), 10% SDS, Proteinase K (15 mg/mL, MC500B Promega), and phenol:chloroform:isoamyl alcohol (25:24:1). The upper phase was extracted twice with phenol:chloroform:isoamyl alcohol (25:24:1) followed by further purification with chloroform:isoamyl alcohol (24:1) two times. Standard ethanol precipitation was performed to recover DNA pellets, which were resuspended in 100 µL of Tris-HCL buffer (10 mM, pH 8.0). Fecal DNA from the human donor sample used in this study was similarly extracted and sequenced separately.

### 16S rRNA bacterial gene sequencing

V3-V4 16S rRNA amplicon gene sequencing was performed at the Genomics Core Facility of the University of Nebraska Medical Center using Illumina MiSeq utilizing the 600 cycle v3 MiSeq kit. The following primer sequences were used for the 16S library prep protocol: Forward Primer = 5′TCGTCGGCAGCGTCAGATGTGTATAAGAGACAGCCTACGGGNGGCWGCAG

Reverse Primer = 5′GTCTCGTGGGCTCGGAGATGTGTATAAGAGACAGGACTACHVGGGTATCTAATCC.

### 16S rRNA bacterial gene sequence analysis

Demultiplexed paired-end fastq files received from the sequencing facility were processed at the University of Nebraska Holland Computer Center Crane cluster following the DADA2 (v1.22) pipeline (91). A total of 6,787,923 reads from mouse fecal samples and 415,357 reads from human donor samples were recovered after processing in DADA2. Self-trained naïve Bayes classifier based on SILVA SSU Ref NR 99 138.1 (92) for the V3-V4 region was prepared using RESCRIPt (93) and used for taxonomic classification in QIIME2 (94). New names for the rank of bacterial phylum proposed by the International Committee on Systematics of Prokaryotes (ICSP) were adopted (95, 96). A phylogenetic tree was generated by FastTree analysis (97) using an MAFFT alignment (98) in QIIME2. Unassigned features at the phylum level, features assigned to mitochondria and chloroplast, and features only present in a single sample were removed from the analysis. A total of 6,618,547 reads and 2,082 Amplicon Sequence Variants (ASVs) were obtained after filtration for the mouse samples and used for further microbiome analysis (Fig. S7). Samples were rarified to 90% of the minimum library size without replacement for downstream analysis using different R packages in R (version 4.2.1). Differential abundance analysis was performed using ANCOMBC (v1.6.2) on the non-rarefied data (99). Taxa were considered differentially abundant if the adjusted *P*-value was <0.05 (False Discovery Rate, FDR). To evaluate the proportion of human donor fecal microbiota engraftment in the dHu-BLT mice, the DADA2 output of mouse and human donor samples were combined and processed similarly as described above for taxonomic assignment and filtration. A total of 7,037,798 reads and 2,310 ASVs were obtained from combined mouse and human donor samples, which were rarified to 90% of the minimum library size without replacement, and shared ASVs were then determined.

### Fecal calprotectin measurement

Human fecal calprotectin (S100A8/A9) was measured from dHu-BLT mouse fecal samples in duplicate via ELISA (DY8226-05, DY008B; R&D systems, MN). Fecal samples were collected in 1.5 mL tubes and diluted with 50× of extraction buffer (0.1 M Tris, 0.15 M NaCl, 1.0 M urea, 10 mM CaCl2, 0.1 M citric acid monohydrate, 5 g/L BSA (pH 8.0)). Samples were vigorously vortexed using a vortex mixer to dissolve the fecal pellets in the extraction buffer. Homogenates were transferred into a new tube and further centrifuged for 20 minutes at 10,000 *× g* at 4°C. The supernatant was then used to measure the calprotectin level as per the manufacturer’s instructions.

### Plasma cytokine profiles

To evaluate systemic inflammation, plasma samples were assayed in duplicate using an Inflammation 20-Plex Human ProcartaPlex Panel (Cat# EPX200-12185-901, ThermoFisher Scientific, Waltham, MA). Inflammatory mediators analyzed included the following: Cytokines: granulocyte-macrophage colony-stimulating factor (GM-CSF), interferon (IFN)-α, IFN-γ, interleukin (IL)−1α, IL-1β, IL-4, IL-6, IL-8, IL-10, IL- 12p70, IL-13, IL-17A, and tumor necrosis factor (TNF) α. Chemokines: interferon γ-inducible protein-10 (IP-10)/CXCL10, monocyte chemoattractant protein-1 (MCP-1)/CCL2, macrophage inflammatory protein (MIP)-1 alpha (MIP-1α)/CCL3, and MIP-1 beta (MIP-1β)/CCL4. Cell adhesion and inflammatory response proteins: intercellular adhesion molecule (ICAM)−1, CD62E (E-selectin), and CD62P (P-selectin). Samples were assayed on a Luminex MAGPIX instrument (Luminex Corporation, Austin, TX) according to the manufacturer’s protocol. Analytes out of range (OOR) in more than one sample in any dietary treatment were excluded from the statistical comparison.

### Statistical analysis

Statistical comparison was performed using Welch’s *t*-test if all data passed the Shapiro-Wilk test of normality or Wilcoxon rank sums test if data from at least one group failed the normality test. In the case of beta diversity, both the Permutational Multivariate Analysis of Variance (PERMANOVA) and Permutational Analysis of Multivariate Dispersion (PERMDISP) were used to assess statistical differences between groups with 999 randomizations. All analyses were performed in R (version 4.2.1) except for the Kaplan-Meier analysis, which was performed with GraphPad Prism 5. A value of *P* < 0.05 was considered statistically significantly different.

## Data Availability

The data sets generated during the current study are available in the NCBI Sequence Read Archive (SRA) repository with accession number PRJNA939259. Scripts related to the current study are freely available from the corresponding author upon request.

## SUPPLEMENTAL MATERIAL

Supplemental Figures msystems.01322-23-s0001.docx Figures S1 to S7.

Supplemental Tables msystems.01322-23-s0002.xlsx Tables S1 to S10.

ASM does not own the copyrights to Supplemental Material that may be linked to, or accessed through, an article. The authors have granted ASM a non-exclusive, world-wide license to publish the Supplemental Material files. Please contact the corresponding author directly for reuse.
