# Supplementary material for: High-fat diet feeding exacerbates HIV-1 rectal transmission
Source: mSystems. 2024 Feb 2;9(3):e01322-23. doi: 10.1128/msystems.01322-23 (PMC10949459; doi:10.1128/msystems.01322-23)
Supplement: Supplemental Figures — Figures S1 to S7. [file msystems.01322-23-s0001.docx]

High-fat diet feeding exacerbates HIV-1 rectal transmission.

Saroj Chandra Lohani^a,b^, Amanda E. Ramer-Tait^c,d^, and Qingsheng Li^a,b,#^

^a^School of Biological Sciences, University of Nebraska-Lincoln, Lincoln, NE, United States

^b^Nebraska Center for Virology, University of Nebraska-Lincoln, Lincoln, NE, United States

^c^Department of Food Science and Technology, University of Nebraska-Lincoln, Lincoln, NE, United States

^d^Nebraska Food for Health Center, University of Nebraska-Lincoln, Lincoln, NE, United States

#Address correspondence to Qingsheng Li, [qli@unl.edu](mailto:qli@unl.edu)

Supplemental Figures


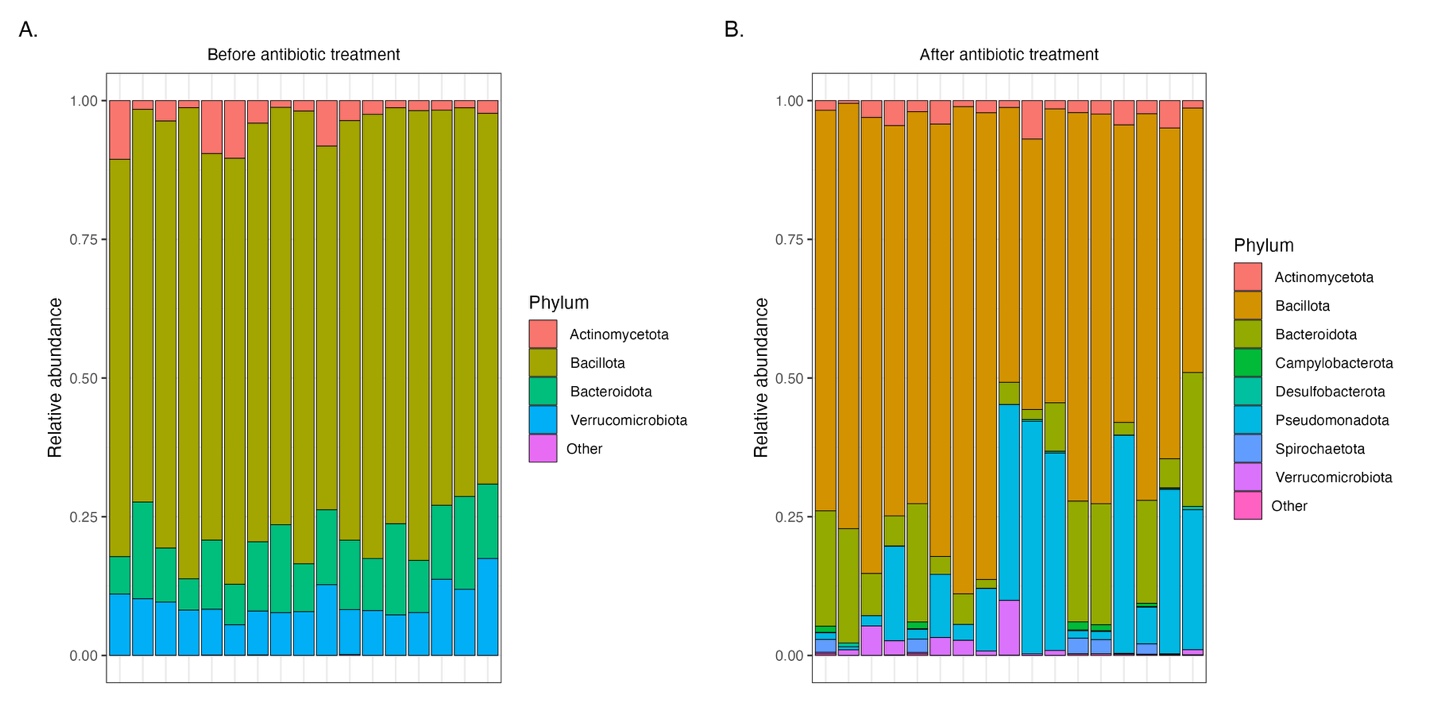


Fig. S1: Gut microbial composition of BLT mice used in the study. The figure illustrates the relative abundance of different phyla in each mouse before antibiotic treatment (A) and after 14 days of antibiotic treatment (B). Each bar on the graph represents an individual mouse. Phylum < 0.005 abundance is grouped as "Other".


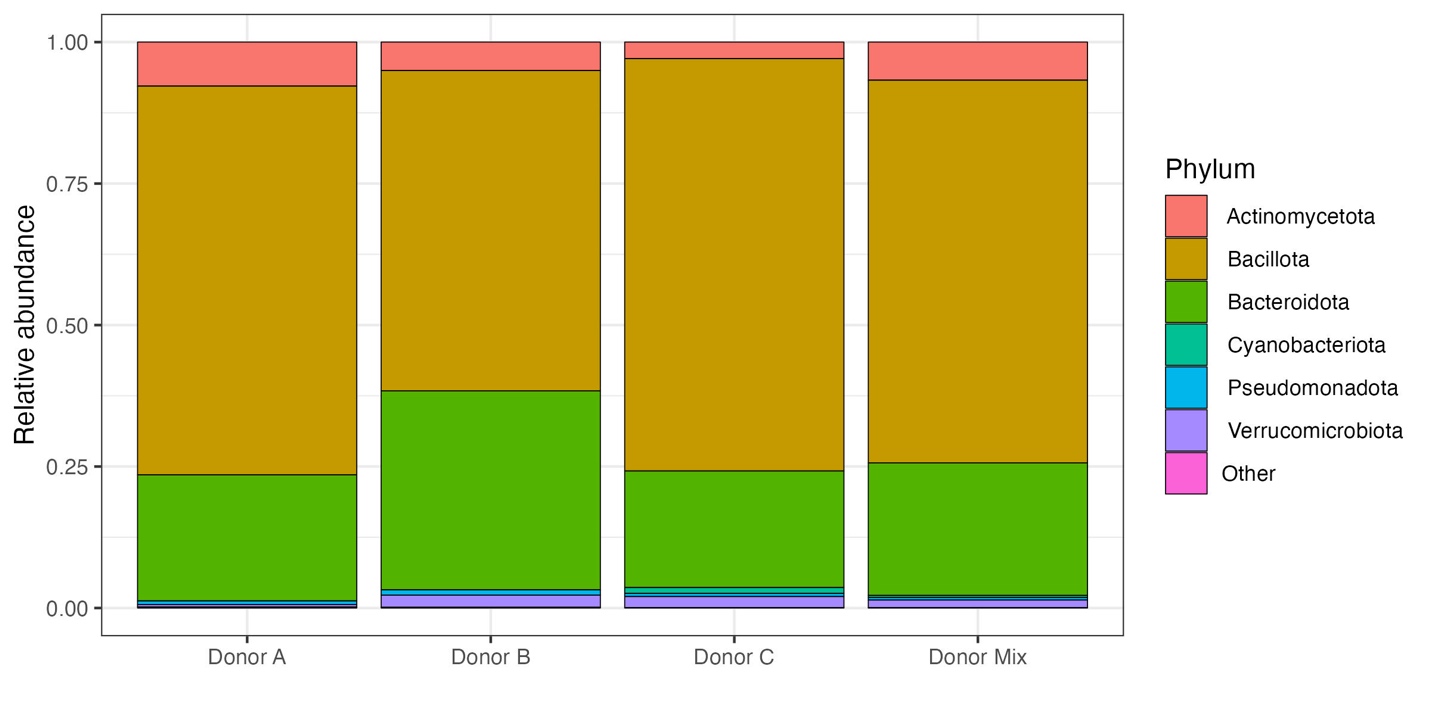


Fig. S2: Microbial composition of donor samples. The figure illustrates the relative abundance of different phyla in each donor sample and donor mix. Each bar on the graph represents an individual donor or donor mix. Phylum < 0.005 abundance is grouped as "Other".


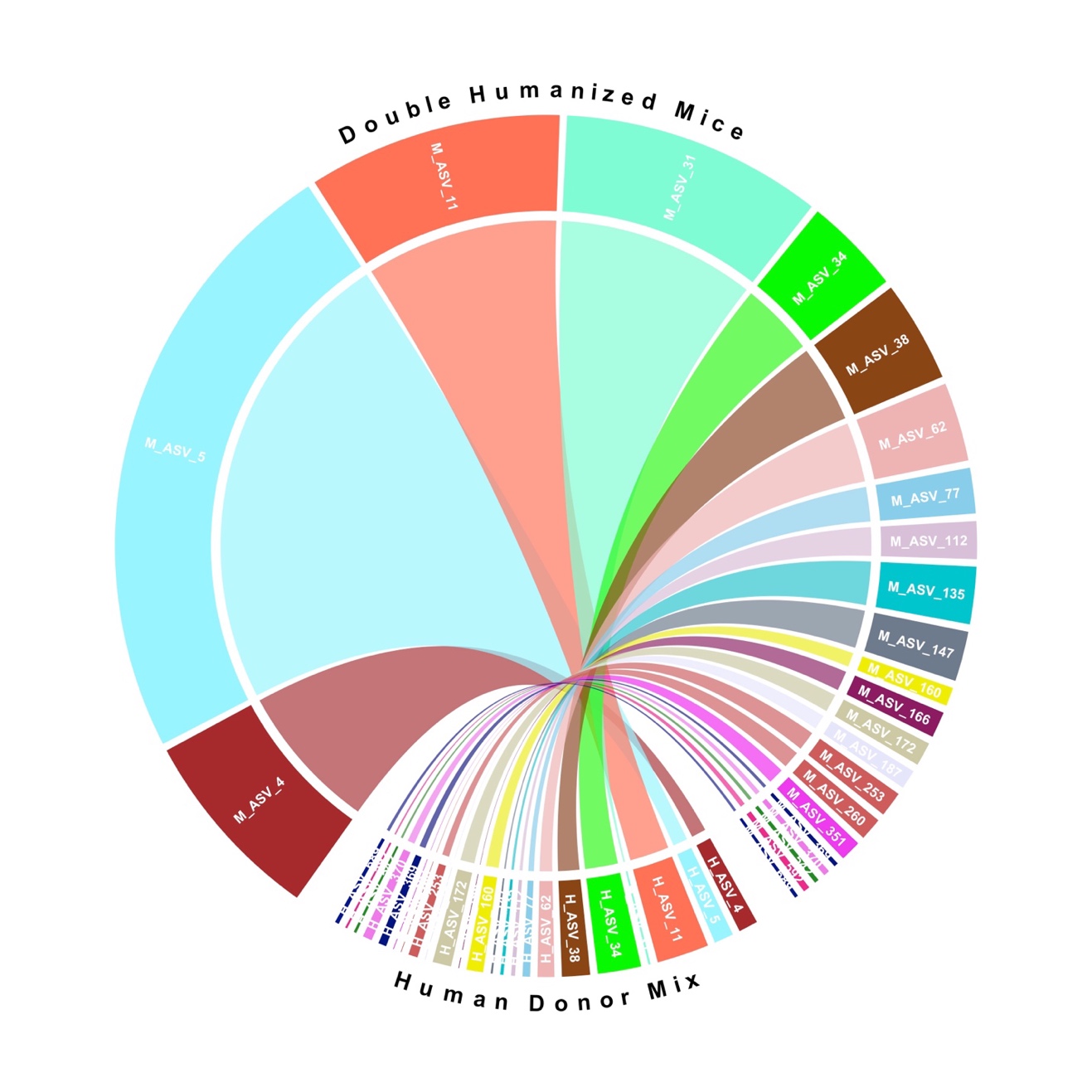


Fig. S3: Human donor mix ASVs present in all dHu-BLT mice one week after FMT. Each sector represents an ASV (“M” represents mouse and “H” represents human donor mix), and the size of the sector corresponds to the mean relative abundance of corresponding ASVs in the dHu-BLT mice and relative abundance in the human donor mix. The width of the ASVs link at each end is proportional to the abundance of respective ASVs in the dHu-BLT mice and the human donor mix.


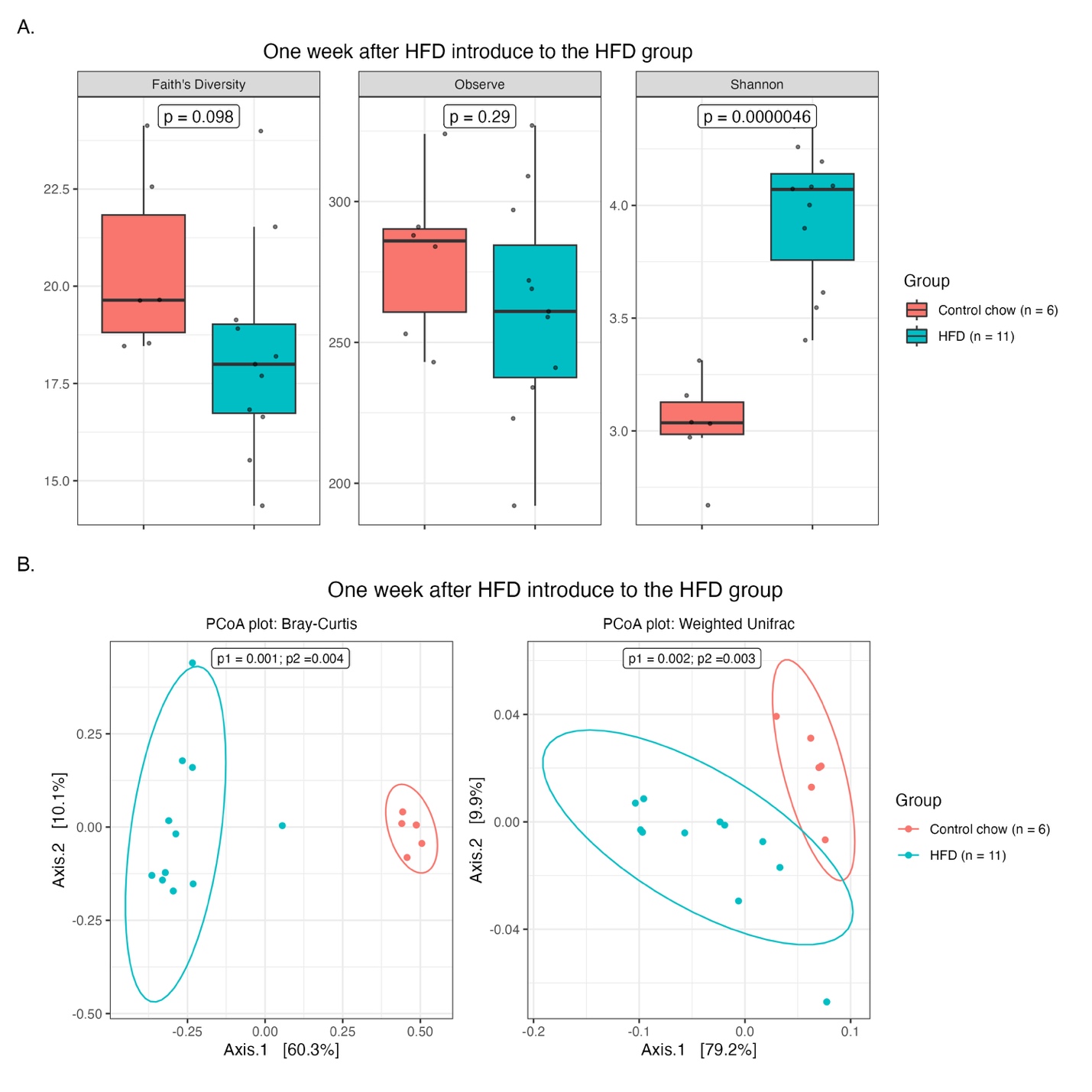


Fig. S4*.* Alpha diversity (A) and beta diversity (B) metrics for mice at one week after HFD feeding was initiated in the HFD group. Each data point represents an individual mouse, p1 = Permutational Multivariate Analysis of Variance (PERMANOVA), and p2 = Permutational Analysis of Multivariate Dispersion (PERMDISP).


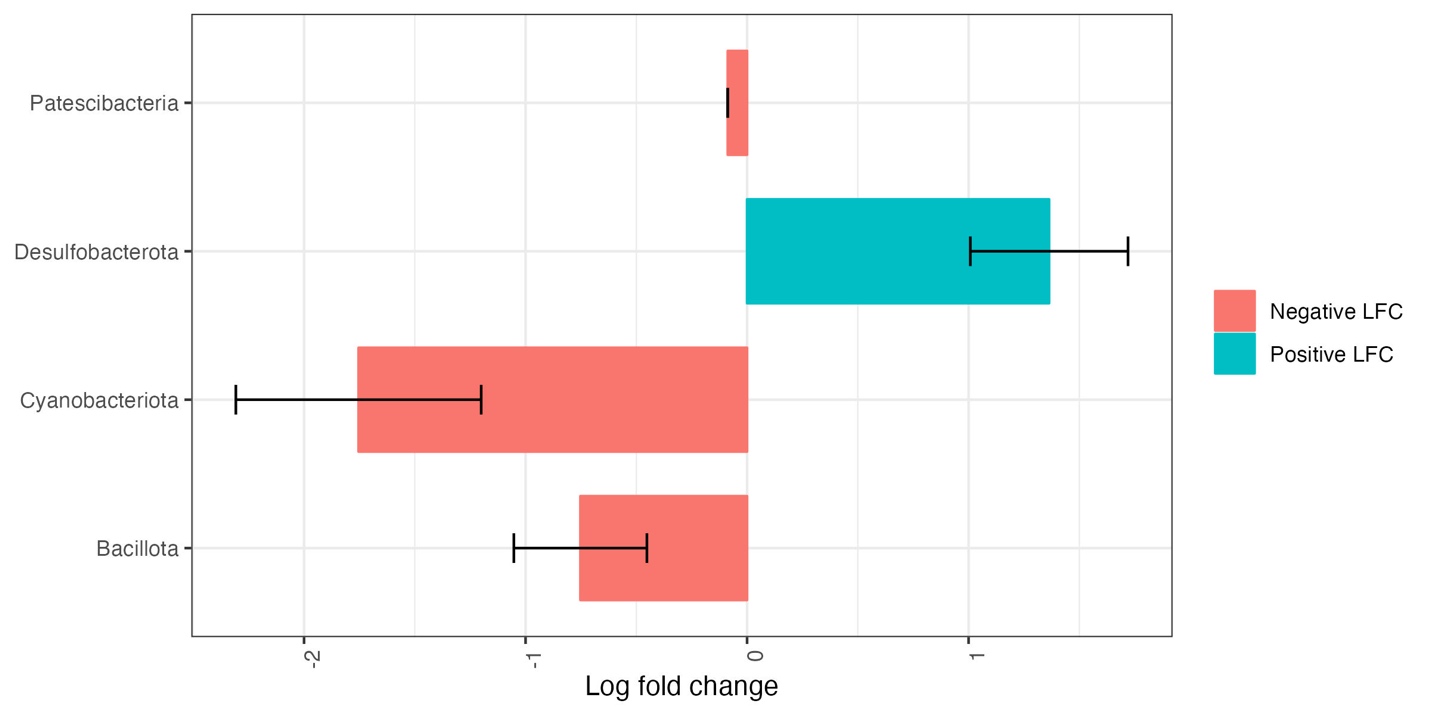


Fig. S5. Waterfall plot of log fold change (natural log) of absolute abundance of differentially abundant taxa at the phylum level. Only significant log-fold changes (with fdr p-value < 0.05) from the result of ANCOM-BC analysis are shown.


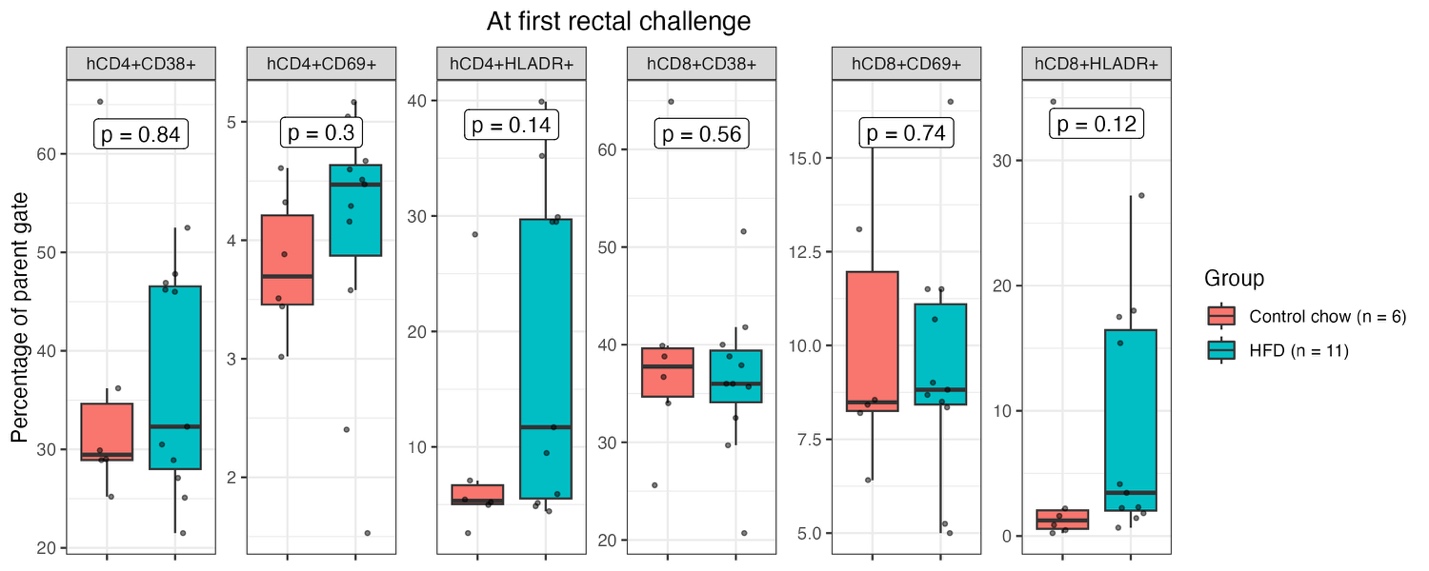


Fig. S6. T-cell activation marker at the first rectal challenge. Each data point represents the individual mouse's respective T-cell activation marker. p-value < 0.05 is considered as significant.


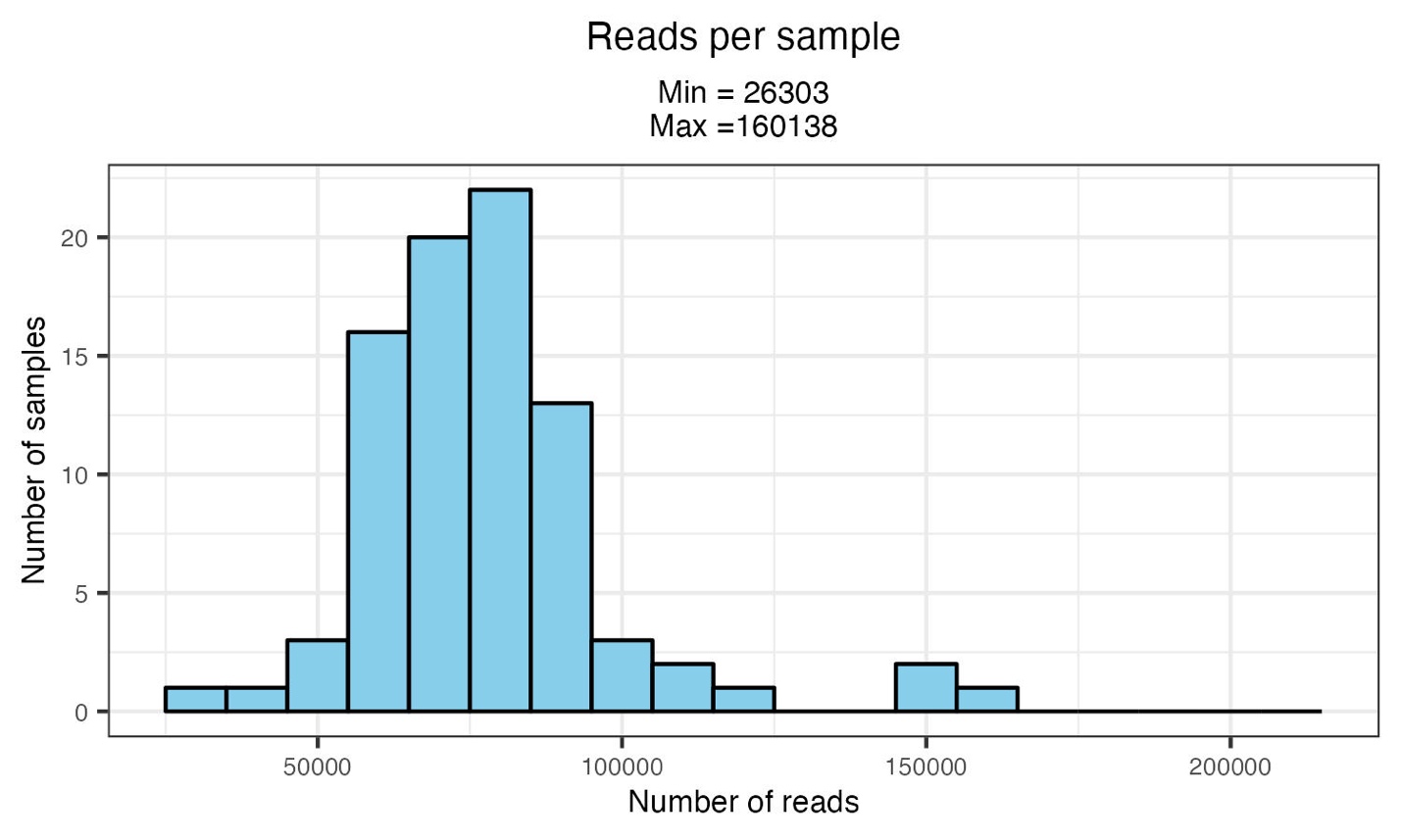


Fig. S7. Reads counts distribution of dHu-BLT mice fecal samples after filtering unassigned features at the phylum level, features assigned to mitochondria and chloroplast, and features present in a single sample.
